# Predicting the allergenicity of legume proteins using a PBMC gene expression assay

**DOI:** 10.1186/s12865-021-00415-x

**Published:** 2021-04-13

**Authors:** Mark Smits, Marjolein Meijerink, Thuy-My Le, André Knulst, Aard de Jong, Martinus Petrus Maria Caspers, Everton Souto Lima, Lilia Babé, Gregory Ladics, Scott McClain, Geert Houben, Kitty Verhoeckx

**Affiliations:** 1 Netherlands Organisation for Applied Scientific Research (TNO), Utrecht, The Netherlands; 2 Department of Dermatology and Allergology, University Medical Center Utrecht, Utrecht, The Netherlands; 3 Center of Translational Immunology, University Medical Center Utrecht, Utrecht, The Netherlands; 4 DuPont Nutrition and Biosciences, Palo Alto, CA USA; 5 DuPont Nutrition and Biosciences, Wilmington, DE USA; 6 Formerly, Syngenta Crop Protection, LLC, 754 Research Triangle Park, Durham, USA

**Keywords:** Food allergy, Allergenicity prediction assay, Allergenic legume protein pairs, Biomarker discovery, Graded allergenic response

## Abstract

**Background:**

Food proteins differ in their allergenic potential. Currently, there is no predictive and validated bio-assay to evaluate the allergenicity of novel food proteins. The objective of this study was to investigate the potential of a human peripheral blood mononuclear cell (PBMC) gene expression assay to identify biomarkers to predict the allergenicity of legume proteins.

**Results:**

PBMCs from healthy donors were exposed to weakly and strongly allergenic legume proteins (2S albumins, and 7S and 11S globulins from white bean, soybean, peanut, pea and lupine) in three experiments. Possible biomarkers for allergenicity were investigated by exposing PBMCs to a protein pair of weakly (white bean) and strongly allergenic (soybean) 7S globulins in a pilot experiment. Gene expression was measured by RNA-sequencing and differentially expressed genes were selected as biomarkers. 153 genes were identified as having significantly different expression levels to the 7S globulin of white bean compared to soybean. Inclusion of multiple protein pairs from 2S albumins (lupine and peanut) and 7S globulins (white bean and soybean) in a larger study, led to the selection of *CCL2*, *CCL7*, and *RASD2* as biomarkers to distinguish weakly from strongly allergenic proteins. The relevance of these three biomarkers was confirmed by qPCR when PBMCs were exposed to a larger panel of weakly and strongly allergenic legume proteins (2S albumins, and 7S and 11S globulins from white bean, soybean, peanut, pea and lupine).

**Conclusions:**

The PBMC gene expression assay can potentially distinguish weakly from strongly allergenic legume proteins within a protein family, though it will be challenging to develop a generic method for all protein families from plant and animal sources. Graded responses within a protein family might be of more value in allergenicity prediction instead of a yes or no classification.

**Supplementary Information:**

The online version contains supplementary material available at 10.1186/s12865-021-00415-x.

## Background

Food allergy is caused by a hypersensitivity response of the immune system to otherwise harmless proteins in food. The food proteins which cause such immune-mediated hypersensitivity responses are called allergens. The allergenicity of a protein is the potential of a protein to cause sensitization and allergic reactions as defined by Verhoeckx et al. [1]. Differences in the allergenic potential of proteins seem to exist. For example, food allergy to peanut is more common than to white bean, for which reports are scarce in literature [2, 3]. In view of the global ecological challenges, one of the major trends in the food industry is to develop products based on alternative food sources. Currently, the allergenicity of novel food proteins cannot be predicted with the available biological test methods [4]. Activation tests with effector cells (e.g. basophils or mast cells) that are currently used to investigate protein allergenicity cannot be used for this purpose because those assays require serum from sensitized patients in order to be performed, which is not available for novel food proteins. The lack of validated and predictive tests hampers the safety assessment and thus the introduction of novel food protein sources. The development of a reliable bio-assay to evaluate the allergenicity of (novel) food proteins is therefore of great importance to lower the risk of introducing strongly allergenic novel foods on the market. Moreover, such methods can potentially lead to a better understanding which factors influence the allergenicity of food proteins.

ImpARAS, a COST Action which focussed on improving the allergenicity risk assessment, published an extensive review discussing the multiple in vitro assays and different cell types (e.g. epithelial cells, dendritic cells and T cells) which can be used for the evaluation of allergens [5]. The different cell types have distinct advantages and disadvantages. T cells and dendritic cells would be preferred because they play an important role in the development of food allergy, but specific issues reduce their applicability. Limited allergen-specific T cells circulate in the peripheral blood of allergic patients and dendritic cells have specific limitations such as donor variability. An in vitro assay using peripheral blood mononuclear cells (PBMCs) from patients is commonly used to evaluate parameters such as gene expression and cytokine production after stimulation [6, 7]. The advantage of PBMCs is that they compromise of multiple immune cells (e.g. monocytes, natural killer cells, lymphocytes, and dendritic cells) associated with allergy, increasing the likelihood to find predictive biomarkers. PBMCs play an important role in the mammalian immune system and are capable of releasing a multitude of cytokines upon activation that are relevant in the allergic response. PBMCs were found to overexpress the *DEFA1, LTF, AQP3, CLC, TLR4, IL1R2* genes [8] and biomarkers such as interleukin 4 (*IL-4*), *IL-5*, *IL-13* and *IL-10* [9].

Peanut and soybean are legumes which together with chickpea, lupines, lentils, beans and peas belong to the *Fabaceae* family. Many allergens from multiple legumes have been identified and are listed in the WHO/IUIS Allergen database. Proteins are entered in the WHO/IUIS Allergen database when specific immunoglobulin E (IgE) binding in the serum of 5 independent allergic donors is found [10]. Allergenicity varies greatly among legumes, which makes legume proteins an interesting group to use as a reference set to study biomarkers for the prediction of weakly and strongly allergenic proteins. Most legume allergens belong to four protein superfamilies; the prolamins and cupins, the profilins and the pathogenesis-related protein family [11]. 7S (vicilin-type) and 11S (legumin-type) globulins belong to the cupin family [12]. The prolamin family contains the 2S albumins, α-amylase inhibitors, and nonspecific lipid transfer proteins [13]. Proteins from the cupin and prolamin family are seed storage proteins which are important diagnostic markers and associated with allergy [14]. Most protein families consist of strongly and weakly allergenic proteins (i.e. the 2S albumin from peanut, Ara h 2, is a strongly allergenic protein while the 2S albumin from white bean has not been indicated as an allergenic protein). Protein pairs (a combination of an allergenic and a homologous non/weakly allergenic protein) of 2S albumins (e.g. 2S albumin from lupin and peanut), and 7S and 11S globulins (white bean and soybean) could be used to investigate biomarkers which could predict allergenicity.

In this study, an in vitro gene expression assay using PBMCs from non-atopic donors was developed in order to measure differentially expressed genes (DEGs) upon stimulation with different weakly and strongly allergenic legume protein pairs. PBMCs from non-atopic donors were used because predictive assays should be as generically applicable as possible. Additionally, results from PBMCs from atopic donors could be influenced by the donor’s sensitization profile rendering the results of the PBMC gene expression assay source specific. Furthermore, PBMCs from atopic donors are likely to show variations in their response because of differences between atopic diseases (e.g. atopic dermatitis, allergic rhinitis, asthma, and food allergy) and comorbidities.

The aim of this study was to develop a predictive generic in vitro allergenicity assay. For this purpose, PBMCs from non-atopic donors were used together with a reference set of weakly and strongly allergenic legume protein pairs.

## Results

### Strongly allergenic proteins induce a different response compared to weakly allergenic proteins

In the pilot experiment, PBMCs were stimulated with a weakly (phaseolin from white bean) and strongly (Gly m 5 from soybean) allergenic protein. DEGs were identified by RNA-seq and differences in gene expression were found between the stimulation with weakly or strongly allergenic 7S globulins. The largest number of DEGs were found when using the highest concentration (50 μg/mL) of white bean and soybean 7S globulins, and resulted in 4379 DEGs. In comparison, the lowest concentrations (10 μg/mL) of white bean and soybean 7S globulins resulted in 919 DEGs.

Generally Applicable Gene-set Enrichment (GAGE) pathway analysis was used to investigate which pathways were associated with the incubation of 7S globulins from white bean and soybean, and control medium. GAGE pathway analysis showed fourteen significantly changed pathways (Table 1). The cytokine-cytokine receptor interaction (16 genes) and hematopoietic cell lineage pathway (8 genes) are immune-related pathways which were highly significant between the highest concentration of white bean (weakly allergenic) and soybean (strongly allergenic) 7S globulins. Furthermore, other significant immune-related pathways which were associated with the DEGs were the IL-17, PI3K, Rap1, PPAR, JAK-STAT, and the chemokine signalling pathway. The largest number of significant pathways was found to be shared between the highest dose of white bean and soybean 7S globulins. Interestingly, no pathways were found to be significantly changed between control medium and the stimulation with the high concentration of the white bean 7S globulin. Based on the results from the pilot experiment, we decided that the PBMC gene expression assay could be a potential assay to differentiate and predict the allergenic potential of (novel) proteins.

**Table 1:** Significant GAGE pathways

Treatment	Soybean (50 μg/mL)	White bean (50 μg/mL)	Soybean (50 μg/mL)
**Control**	White bean (50 μg/mL)	control	control
Cytokine-cytokine receptor interaction	*p* < 0.01	–	*p* < 0.01
Osteoclast differentiation	*p* < 0.01	–	*p* < 0.01
Neuroactive ligand-receptor interaction	*p* < 0.01	–	*p* < 0.01
Hematopoietic cell lineage	*p* < 0.01	–	*p* < 0.01
Rap1 signalling pathway	*p* < 0.01	–	*p* < 0.01
Chemokine signalling pathway	*p* < 0.01	–	*p* < 0.01
PI3K-Akt signalling pathway	*p* < 0.01	–	*p* < 0.01
Complement and coagulation cascades	–	–	*p* < 0.01
Jak-STAT signalling pathway	*p* < 0.01	–	*p* < 0.01
IL-17 signalling pathway	*p* < 0.01	–	*p* < 0.01
PPAR signalling pathway	*p* < 0.01	–	*p* < 0.01
Ovarian steroidogenesis	*p* < 0.01	–	*p* < 0.01
Focal adhesion	*p* < 0.01	–	–
Protein digestion and absorption	*p* < 0.01	–	–

Differences between the weakly allergenic 7S globulin from white bean and the strongly allergenic 7S globulin from soybean for three comparisons (soybean 50 μg/mL versus white bean 50 μg/mL, white bean 50 μg/mL versus control and soybean 50 μg/mL versus control) are shown. Significance per pathway and comparison is indicated by *p* < 0.01 (unadjusted *p*-value)

### *CCL2*, *CCL7*, *RASD2*, and *IL-24* are potential biomarkers for allergenicity prediction

**Fig. 1:**
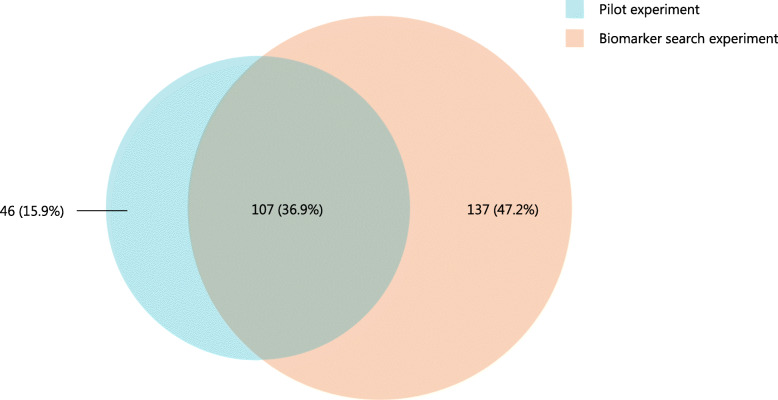
Number of DEGs in the pilot (blue) and biomarker search (orange) experiment. Overlap between the pilot and the biomarker search experiment was found for 107 (36.9%) DEGs. DEGs were selected based on their significance (p adj. < 0.1) and log_2_ fold change (> 0.32)

2S albumins from lupine and peanut and the 7S globulins from white bean and soybean were selected to investigate potential biomarkers for allergenicity in a biomarker search experiment. The 2S albumin from lupine (δ-conglutin) and the 7S globulin from white bean were categorized as weakly allergenic, the 2S albumin from peanut (Ara h 2) and the 7S globulin from soybean were categorized as strongly allergenic. PBMCs from non-atopic donors were stimulated with the different 2S albumin and 7S globulin protein pairs and gene expression was investigated. The biomarker search experiment confirmed the results of the pilot experiment. DEGs were regarded as significant when the *p* adjusted value was < 0.1 and the log_2_ fold change was > 0.32. 107 DEGs (36.9%) were found to be shared between the 7S globulins from soybean and white bean (50 μg/mL) in the pilot and the biomarker search experiment (Fig. 1). The percentage of genes which were not found to be shared between the pilot and biomarker search experiments can be explained by multiple factors such as intra-donor differences, chance, and noise. Certain biomarkers were slightly over the log_2_ cut-off value in the biomarker search experiment and slightly below the log_2_ cut-off value in the pilot experiment. However, a large number of shared DEGs between independent experiments strengthened the reliability of the PBMC gene expression assay.

**Fig. 2:**
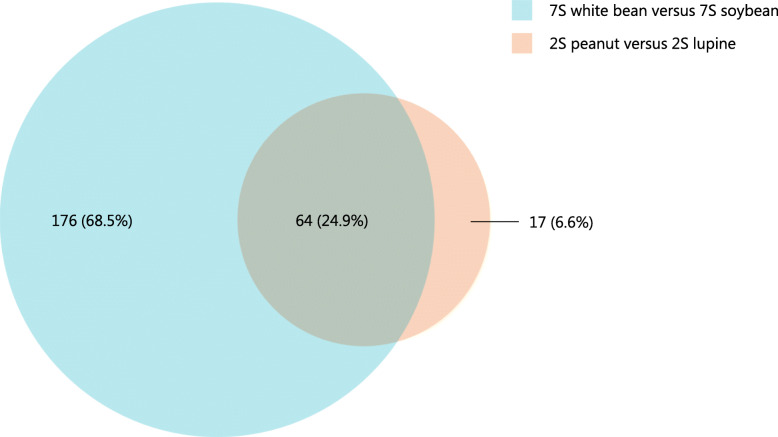
Number of overlapping DEGs between 2S albumins and 7S globulins. The orange circle indicates the DEGs from the comparison of 2S albumins from lupine and peanut (81 genes in total) and the blue circle between 7S globulins from white bean and soybean (240 genes in total). Genes were selected as DEGs if the p adj. was < 0.1 and the log_2_ fold change was > 0.32. 64 genes were overlapping between the 7S globulins and 2S albumins comparisons

Overlapping DEGs between the different legume protein pairs are shown in Fig. 2. In total, 64 DEGs were shared by the 2S albumins from peanut and lupine, and the 7S globulins from soybean and white bean. The details of these DEGs can be found in Additional files (see Additional file 1: Table 1). The results indicate that the proteins which are considered to be strongly allergenic elicit a comparable reaction from the PBMCs, which differs considerably from the weakly allergenic proteins. GAGE pathway analysis was performed on these 64 DEGs. Again, the cytokine-cytokine receptor interaction (ten genes) and TGF-beta signalling pathway (three genes) and chemokine signalling pathway (four genes) were significantly associated with the DEGs. *CCL2*, *CCL7*, *IL-24*, and *RASD2* were selected as biomarkers based on their log_2_ fold change (> 0.32), significance (*p* adj. value), biological relevance, and expression levels for validation by Real Time Quantitative PCR (qPCR).

**Fig. 3:**
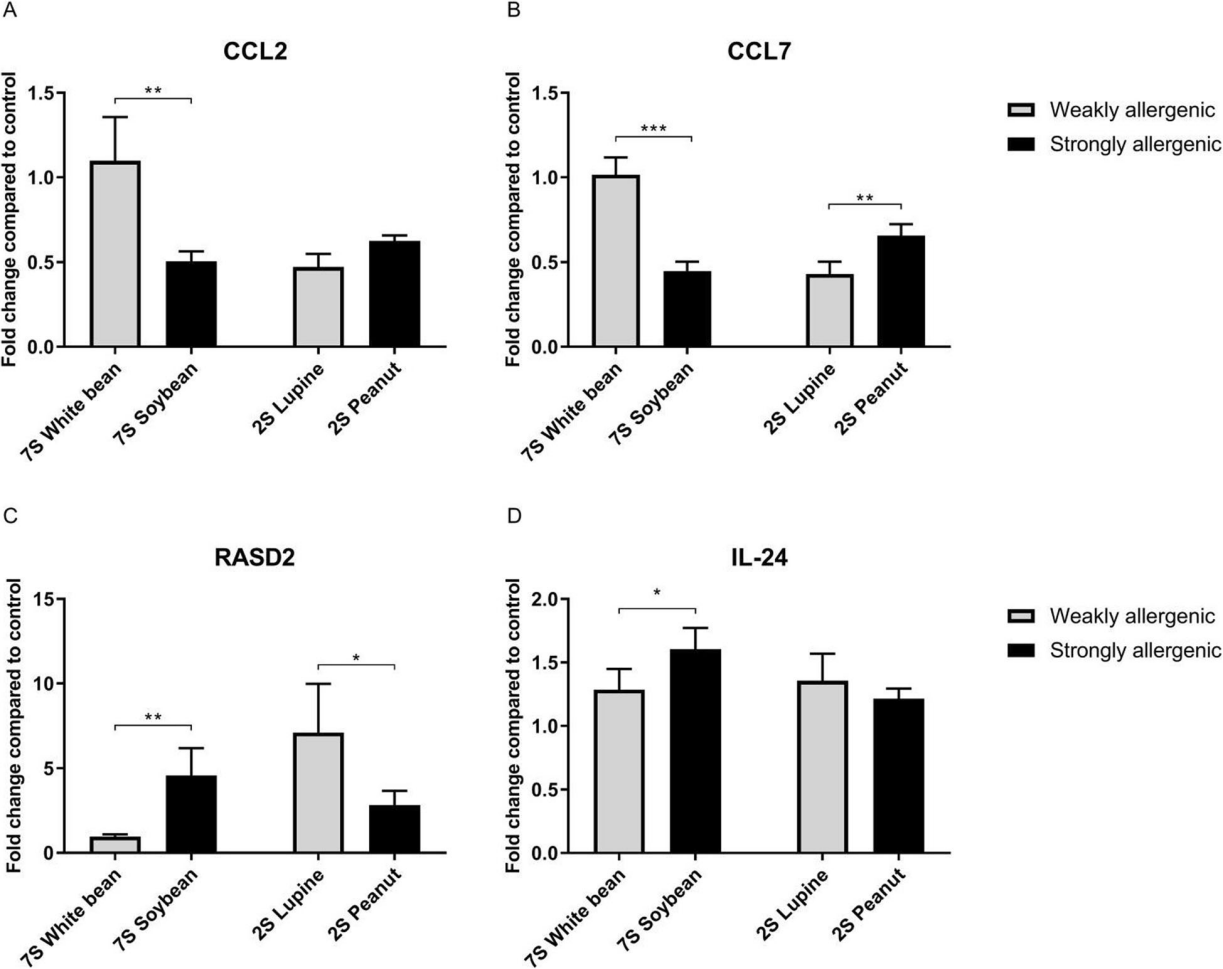
qPCR data for CCL2 (**a**), CCL7 (**b**), RASD2 (**c**), and IL-24 (**d**) compared to control (unstimulated cells). The displayed results are the average of five donors, of which the -ΔΔCt was calculated from five replicates. Data is presented as mean ± standard deviation (SD) and analysed by an independent t-test. A *p*-value of < 0.05 was considered statistically significant and relevant comparisons are shown. The number of symbols indicates the level of significance: * *p* < 0.05, ** *p* < 0.005, *** *p* < 0.0005

The RNA-sequencing (RNA-seq) results were confirmed by qPCR as shown in Fig. 3. For the 7S proteins, an increase in *CCL2* (Fig. 3a) and *CCL7* (Fig. 3b) expression was seen which was significantly lower for the strongly allergenic protein compared to the weakly allergenic protein. Increased expression of *RASD2* (Fig. 3c) and *IL-24* (Fig. 3d) was seen which was significantly higher for the weakly allergenic protein compared to the strongly allergenic protein. For the 2S proteins, an increase in *CCL2, CCL7, RASD2*, and *IL-24* expression was seen. *CCL7* expression was significantly higher for the strongly allergenic protein and RASD2 expression was significantly lower for the strongly allergenic protein compared to their weakly allergenic counterpart. The selected biomarkers seemed to be also able to distinguish significantly between weakly and strongly allergenic proteins within the 7S and 2S protein family. The expression of these genes was further investigated using a larger panel of weakly and strongly allergenic legume proteins.

### Graded gene expression (*CCL2*, *CCL7* and *RASD2*) responses were seen for multiple legume proteins

**Fig. 4:**
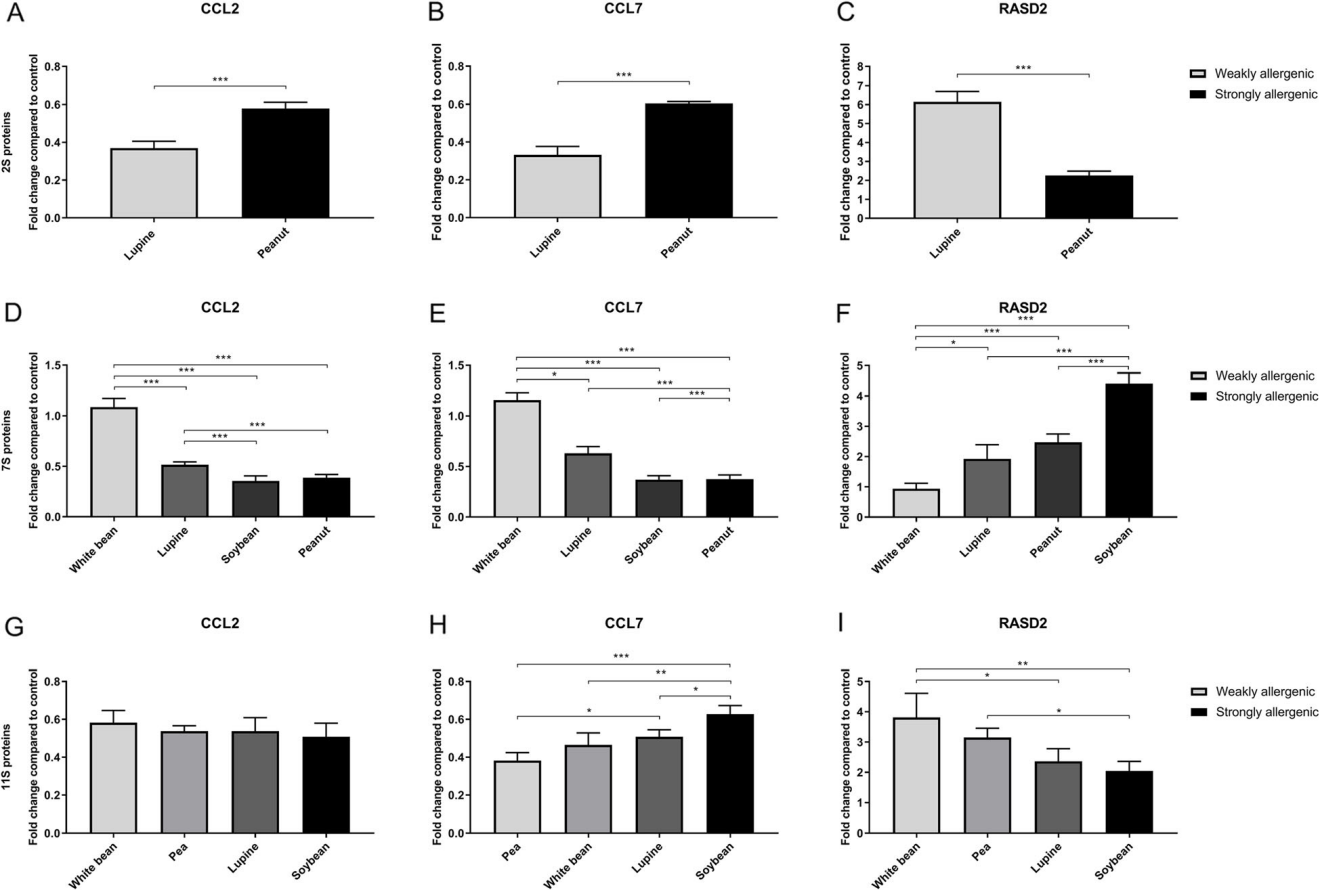
qPCR data for CCL2 (**a, d, g**), CCL7 (**b, e, h**), and RASD2 (**c, f, i**) compared to control (unstimulated cells). The displayed results are the average of one donor, of which the -ΔΔCt was calculated from four replicates. Fold changes for CCL2, CCL7, and RASD2 compared to control (unstimulated cells) are shown. Data is presented as mean ± SD and analysed by an independent t-test or one-way analysis of variance (ANOVA) followed by Tukey’s multiple comparison test. A *p*-value of < 0.05 was considered statistically significant. The number of symbols indicates the level of significance: * *p* < 0.05, ** *p* < 0.005, *** *p* < 0.0005

In a third experiment, the selected biomarkers from the biomarker experiment were validated by stimulation of PBMCs with a large panel of weakly and strongly allergenic 2S albumins, and 7S and 11S globulins. Figure 4 shows the results of the qPCR analysis for *CCL2*, *CCL7*, and *RASD2* using this larger panel of proteins. Incubation with the weakly allergenic 2S albumin from lupine (δ-conglutin) resulted in a significantly lower expression of *CCL2* (Fig. 4a) and *CCL7* (Fig. 4b) compared to the allergenic 2S albumin from peanut (Ara h 2). Expression of *RASD2* (Fig. 4c) showed an opposite expression pattern and expression was significantly higher after incubation with δ-conglutin compared to Ara h 2.

In case of the 7S globulin proteins, significant differences were found for *CCL2*, *CCL7* and *RASD2*. Incubation with the weakly allergenic 7S globulin from white bean (phaseolin) resulted in a significantly higher expression of *CCL2* (Fig. 4d) and *CCL7* (Fig. 4e) compared to the 7S globulins from lupine (Lup an 1), peanut (Ara h 1) and soybean (Gly m 5). Additionally, incubation with the 7S globulin from lupine resulted in a significantly higher expression in *CCL2* compared to soybean and peanut. This is also seen in the expression of *CCL7*, which was significantly lower in PBMCs stimulated with the 7S globulin from soybean and peanut compared to the 7S globulin from lupine. *RASD2* (Fig. 4f) expression was significantly lower for the 7S globulin from white bean compared to the 7S globulins from lupine, peanut and soybean.

The results for the 11S proteins are less pronounced. Expression of *CCL2* (Fig. 4g) was not significantly different between weakly and strongly allergenic 11S globulins. Significant differences were seen for *CCL7* (Fig. 4h) and *RASD2* (Fig. 4i) expression between weakly allergenic 11S globulins from white bean (legumin) and pea (legumin A) and the strongly allergenic 11S globulin from soybean (Gly m 6). The difference between soybean and lupine (α-conglutin) was not as pronounced, although it was significant for *CCL7* expression.

*IL-24* did not seem to be able to significantly differentiate between weakly and strongly allergenic proteins (data not shown). Incubation with the different proteins generally showed graded responses suggesting graded biological activities.

## Discussion

This study shows that an in vitro PBMC gene expression assay was able to distinguish weakly from strongly allergenic 2S and 7S legume proteins based on the expression of *CCL2*, *CCL7*, and *RASD2*. This was seen to a lesser extent for 11S globulins. The results were confirmed in three independent experiments using two analytical techniques (RNA-seq and qPCR), which makes the results reliable.

Developing an assay to investigate the allergenic potential of food proteins is currently the focus of many research groups as reviewed by ImPARAS, a COST Action focussing on the improvement of allergenicity risk assessment strategy (www.imparas.eu) [4]. However, none of these studies were conducted using a large set of weakly and strongly allergenic proteins from different protein families. Studying the allergenicity of food proteins is complex and many in vitro models and cell types can be used. Therefore, information regarding which way the development could potentially head is important. Our study has shown that the PBMC gene expression assay was able to differentiate between weakly and strongly allergenic legume proteins within a protein family (2S albumins and 7S globulins). The PBMC fraction contains lymphocytes (T cells, B cells, natural killer cells), monocytes, and dendritic cells, so a mixture of signals from the different cells can be expected. From the biomarkers that we found, we may conclude that the monocyte fraction of the PBMCs is most likely responsible for the discovered DEGs due to the up- or downregulated cytokine and chemokine genes and the GAGE pathway analysis. GAGE pathway analysis showed that significant DEGs in the pilot and biomarker search experiment were associated with immunologically relevant pathways (cytokine-cytokine receptor interaction, IL-17, PI3K, Rap1, PPAR, JAK-STAT, and the chemokine signalling pathway [15–21]). RASD2, IL24, CCL2 and CCL7 are immunologically relevant biomarkers in allergy. CCL2 and CCL7 were reported to be involved in Th_2_ polarization [22, 23] and RASD2 activates the mTORC1 pathway which plays an important role in mast cell cytokine production, growth and survival [24, 25]. The immunological role of the suggested biomarkers in mast cell activation and food allergy is summarized in Additional Figure 1. Mast cells are an important effector cell in food allergy and the discovered biomarkers play an important role in regulation and activation of mast cells. Based on the pilot and biomarker search experiments, *CCL2*, *CCL7*, *IL-24*, and *RASD2* were selected to be further investigated using RNA-sequencing and qPCR. *IL-24* expression was not able to distinguish between weakly and strongly allergenic proteins and was therefore omitted as a potential biomarker. Strongly allergenic 7S globulins reduced expression of *CCL2* and *CCL7* compared to weakly allergenic 7S globulins. Incubation with strongly allergenic 2S albumins gave a reversed response. The reason for this result is not clear to us. Possibly a mixture of reactions in the different cells present in PBMC fraction may account for this or proteins from different protein families induce allergies via different metabolic routes and receptors. This might also explain why no significantly differences were found for these biomarkers for the 11S globulin protein pairs.

The results from the PBMC gene expression assay showed graded responses to allergens within a protein family, which poses the question whether we should use ranking of proteins according to their allergenic potency (weak, intermediate to strong allergenic) instead of categorising them as allergenic or non-allergenic in a yes or no classification. This might improve the outcomes of current immunological assays for allergenicity prediction. In this respect, it is interesting to compare the graded responses of our study with the ranking of (allergenic) proteins based on clinical data. Previously, the prevalence of sensitization and allergic symptom elicitation potency were proposed to be used as parameters for expressing and comparing allergenicity of foods by the ImpARAS consortium [4]. However, studies evaluating sensitization to multiple legumes simultaneously are scarce. In a Portuguese study, sensitization in legume-allergic patients was most commonly seen for peanut (71%), followed by lupine (60%), soybean (50%), and white bean (36%) [26]. Unfortunately, these results are based on IgE binding to total protein extracts, and not to individual proteins. The 7S globulin from peanut (Ara h 1) could, for instance, be less allergenic than the 7S from soybean (Gly m 5) even though the total extract of peanut is more allergenic then the total extract from soybean. The expression of *CCL2*, *CCL7* and *RASD2* seems to follow the rankings based on the prevalence of sensitization with clear differences between weakly allergenic white bean and lupine and strongly allergenic peanut and soybean.

In our research, PBMCs from non-atopic donors were used for the development of an predictive assay. PBMCs from atopic donors would be preferred because allergic responses are more likely to develop in individuals with an atopic predisposition. However, a pilot experiment with PBMCs from atopic donors which were stimulated with 7S globulins from soybean and white bean showed no significant differences between the weakly or strongly allergenic 7S globulins due to inter-donor differences. The inter-donor variation hampered the development of a predictive assay and can likely be attributed to differences in clinical responses between atopic donors (severity of allergy and comorbidities). In addition, the difference in composition of the PBMC fraction between the donors may also be associated with the inter-donor differences that were seen. It was therefore decided to continue with PBMCs from non-atopic donors. However, the use of PBMCs from atopic donors should not be excluded in advance when identifying predictive biomarkers for other protein families. Furthermore, evaluation of the selected biomarkers using PBMCs isolated from atopic and allergic donors should be performed in future studies to further validate the biomarkers.

The goal of our study was to find biomarkers that could distinguish strongly allergenic food proteins from weakly allergenic proteins. Biological validation of the discovered biomarkers must therefore be performed as well, because regulators and policy makers will likely require more information regarding the immunological role of the discovered biomarkers in atopic diseases before these biomarkers can be implemented in the current allergenicity risk assessment strategy. Biological validation of the selected biomarkers will therefore aid in acceptance and implementation. Additional Figure 1 shows that the discovered biomarkers are indeed associated with specific effector cells (mast cells) and immunological roles (Th_2_ polarization). The immunological role of the selected biomarkers should be further explored by performing functional assays with the selected allergens to investigate their role in Th_2_ polarization and basophil or mast cell activation.

Based on which protein family was used to stimulate the PBMCs, a difference in the direction of up- or downregulation was found, which indicates that different proteins families may have distinct reaction mechanisms. Therefore it might be ambitious and even challenging to find a predictive assay which is applicable to a multitude of protein families from multiple protein sources due to the differences in intrinsic properties of the proteins. Furthermore, the impact of food processing and matrix could not be evaluated due to the use of purified proteins. Processing resulted in loss of proteins of interest to the cooking water and in aggregation which made purification increasingly difficult. The presence of toxic substances such as lipopolysaccharide (LPS) hampered the evaluation of matrix effects.

## Conclusions

The PBMC gene expression assay may have the potential to distinguish between weakly and strongly allergenic legume proteins. However, the method is not generic for all protein families. We suggest that future assay development should focus on developing a method to predict the allergenicity of proteins within a protein family and not a generic assay for all proteins and sources. Secondly, the possibility to use graded response instead of yes or no allergenicity classification should be investigated. Thirdly, the monocyte and macrophage fraction is likely to be responsible for the discovered DEGs, due to the discovered biomarkers and associated pathways, which makes them an interesting cell type to further investigate for the development of a predictive allergenicity assay.

## Methods

### Isolation of legume allergens

Weakly and strongly allergenic proteins were extracted using the Osbourne extraction in a procedure adapted from Freitas et al. [27]. In brief, healthy legume seeds were grinded to flour and sieved afterwards to remove lumps. After defatting by a Soxhlet extractor using petroleum ether for 24 h, albumins were extracted using an aqueous buffer (10 mM CaCl_2_, 10 mM MgCl_2_, pH 8). The suspension was centrifuged at 9000 rpm for 40 min at 7 °C (Lynx F9-6 × 1000 Lex rotor). The supernatant containing the albumin was collected. The pellet containing the globulins was suspended in 10 mM CaCl_2_, 10 mM MgCl_2_, (pH 8) and centrifuged again for 40 min at 7 °C. The supernatant was discarded and the pellet containing the globulins was extracted using a high salt buffer (100 mM Tris/HCl, 1 M NaCl, 10 mM EDTA and 10 mM EGTA, pH 8.2). The suspension was again centrifuged for 40 min at 7 °C. The supernatant containing the globulin fraction was collected. The globulin fraction was divided in a 2S albumin fraction and a 7S and 11S globulin fraction by 100 kDa ultra-filtration. The individual proteins were further purified using anion-exchange chromatography under LPS free conditions after ammonium sulphate fractionation. Ammonium sulphate was added to the globulin fraction in a final concentration of 60% or 70%. After centrifugation (20 min at 9000 rpm), the supernatants were separated from the ammonium sulphate pellets. 3 mL of the supernatant was desalted using P10 columns equilibrated and eluted with 20 mM Tris/HCl and 100 mM NaCl (pH 8.2). Pellets were solubilized in 20 mM Tris/HCl and 100 mM NaCl (pH 8.2). The fractions were stored at − 20 °C until further purification.

The following individual proteins were purified from: white bean (phaseolin and legumin), soybean (Gly m 5 and Gly m 6), lupine (α-conglutin, δ-conglutin, and Lup an 1), peanut (Ara h 1, Ara h 2), and pea (legumin A). The samples (2.5 g protein) in buffer A (20 mM Tris/HCl, pH 8) were loaded on the column (Source 15Q™, GE Healthcare) and eluted using a linear gradient from 0 to 60% buffer B (20 mM Tris/HCl + 1.0 M NaCl, pH 8) for 40 min (40 mL/min), followed by a linear gradient from 60 to 100% B for 4 min. The protein purity of all proteins was > 95%, except for Gly m 5 (> 80%), α-conglutin (> 85%), and legumin A (> 90%) and was measured using LC-MS/MS, LC-UV and SDS-PAGE. The identity of all proteins was confirmed using these techniques. Proteins were categorized as strongly allergenic when they were listed as allergens in the WHO/IUIS Allergen database. An overview of the sources, protein family, and allergenicity based on presence in the WHO/IUIS Allergen database of the allergens is given in the Additional files (see Additional file 2: Table 2). Proteins are entered in the WHO/IUIS Allergen database when specific IgE binding in the serum of 5 independent allergic donors is found [10]. LPS content of the proteins was assessed by EndoZyme® II Recombinant Factor C Assay and proteins were considered to be LPS-free (< 0.25 EU/mL) based on the guidelines of the US Food & Drug Administration. Furthermore, no TNF response was detected after stimulation of RAW cells (macrophage cell line) with the legume proteins (data not shown).

### Isolation of PBMCs

PBMCs were isolated from buffy coats from donors from Sanquin (Sanquin Blood Bank, The Netherlands) by Ficoll-Paque™ in 50 mL Leucosep™ tubes. The sera of the donors were checked for their atopic status at the University Medical Center Utrecht by evaluation of specific antibodies in blood. Non-atopic donors were selected based on negative responses (< 0.35 kU/L) to an inhalation (ImmunoCAP Phadiatop (d1, e1, e5, tx9, gx3, wx3, mx1) and food allergen mix (fx5e). The PBMCs were washed 3–6 times with PBS with 2% heat-inactivated foetal calf serum (FCS) and after centrifugation, the cell pellet was reconstituted in culture medium (RPMI 1640) supplemented with 10% heat inactivated FCS, 1% Ultraglutamin and 1% penicillin/streptomycin. Cells were counted and freezing medium (culture medium containing 40% FCS and 10% dimethyl sulfoxide) was added to prepare a cell suspension of 50–150 × 10^6^ cells/mL (Beckman Coulter™). Cells were stored in a liquid nitrogen tank until further usage. Three experiments were performed with PBMCs from non-atopic donors which were incubated with 2S albumins, and 7S and 11S globulins. PBMCs from four donors were stimulated with 10 μg/mL or 50 μg/mL 7S globulins from white bean (phaseolin) and soybean (Gly m 5) in the pilot experiment (five replicates per donor). In the biomarker search experiment, PBMCs from five donors were stimulated with 50 μg/mL 2S albumins from lupine (δ-conglutin) and peanut (Ara h 2), and 7S globulins from white bean and soybean (five replicates per donor). In the third PBMC experiment, PBMCs from one donor were used and stimulated with 50 μg/mL 2S albumins, and 7S globulins (phaseolin, Gly m 5, Ara h 1, and Lup an 1) and 11S globulins (legumin, Gly m 6, legumin A, and α-conglutin) from white bean, soybean, pea, lupine and peanut (four replicates). Based on the results from the pilot and biomarker search experiment, four replicates was deemed to be acceptable for statistical analysis.

### PBMC stimulation and RNA isolation

Frozen PBMCs were rapidly thawed in a water bath of approximately 37 °C and transferred to 15 mL tubes containing culture medium. After centrifugation and removal of DMSO, the cells were suspended in culture medium. The PBMCs were plated into sterile 96-well plates (Corning™) in a concentration of 1 × 10^5^ cells per well and placed in a humidified incubator (37 °C, 5% CO_2_) until exposure. The weakly and strongly allergenic proteins, including medium and LPS controls were prepared and added to the PBMCs in the 96-well plates. All weakly and strongly allergenic proteins (in 20 mM Tris + 0.2 M NaCl, pH 8.0) were diluted with culture medium and 100 μL/well was added to the 96-well plates. The PBMCs were stimulated by exposure in a humidified incubator (37 °C, 5% CO_2_) for four hours and afterwards centrifuged at 400 x *g* for five minutes. Supernatant was discarded and cells were collected in lysing buffer from the NucleoSpin® RNA isolation kit (Machery Nagel™). The RNA isolation was performed according to the manufacturer’s protocol. RNA quality was assessed using fragment analysis using the DNF-471-500 standard sensitivity RNA kit from Agilent™. RNA quality number was determined with Prosize 2.0 (Data Analysis software 2.0.0.51).

### RNA analysis

RNA content was then analysed by RNA-seq according to the manufacturer’s protocol by GenomeScan B.V. (Leiden, The Netherlands). The NEBNext Ultra Directional RNA Library Prep Kit for Illumina was used to process the samples. In brief, mRNA was isolated from total RNA using the oligo-dT magnetic beads. The quality and yield after sample preparation was measured with the Fragment Analyzer. Clustering and DNA sequencing using the Illumina NextSeq 500 was performed according to manufacturer’s protocols using a concentration of 1.6 pM of DNA.

### Viability and functionality of PBMCs

Functionality and viability of the PBMCs was tested at the start of the stimulation experiments. Functionality was assessed by exposing the PBMCs (1 × 10^5^ cells per well) to culture medium (negative control) and to LPS (1 μg/mL) as a positive control for PBMC activation. After 24 h of incubation the plates were centrifuged at 400 x *g* for five minutes. 150 μL of supernatant was transferred to Micronic tubes for TNF-α analysis to assess functionality of the PBMCs. TNF-α was measured in the cell supernatants using a TNF-α ELISA kit from Life Technologies according to the manufacturer’s protocol. PBMC viability was determined using flow cytometry (MACSQuant analyzer 10) after addition of propidium iodide and four hours incubation at 37 °C. Both the functionality and viability of the PBMCs were considered to be sufficient (data not shown). LPS (1 μg/mL) stimulation was used as a control for PBMC activation and induced TNF-α (> 1200 pg/mL) release which was significantly higher compared to culture medium stimulation as a negative control (< 100 pg/mL). Furthermore, cell death did not exceed 15%.

### Statistical and pathway analysis of RNA-seq data

RNA-seq data was analysed using DESeq2. This program performed a RNA-seq quality control, by visualisation of correlation matrices and principal component analysis, for the detection of outliers. In addition, detection of significant DEGs for comparisons of two groups of samples was performed based on the *p* adjusted (*p* adj.) value. The *p* adj. value was based on the method developed by Benjamini and Hochberg and is commonly used when analysing large data sets [28]. The *p* adj. value of 0.1 is a corrected *p*-value for the false discovery rate and the expected proportion of false discoveries amongst the rejected hypotheses. Though a *p*-value of 0.05 is commonly used, the *p*-value of 0.1 after correction using the Benjamini and Hochberg method is comparable and even stricter than an observed *p*-value of 0.05. This stricter cut-off was used because RNA-seq results in a large number of tests for > 10.000 genes and this must be corrected. GAGE pathway analysis was performed using the Kyoto Encyclopedia of Genes and Genomes pathway database.

### Validation of selected biomarkers using qPCR

For gene expression analysis, the QuantiTec Multiplex kit (Qiagen™) was used. Specific Taqman probes and primers were designed. 3-plex qPCR was carried out in the presence of ROX, a passive reference dye. Transcripts of the genes of interest were obtained via www.ensemble.org. A single gene can produce multiple different RNAs (i.e., transcripts) Geneious 7.0.6 was therefore used to align the transcripts. Primer and probes were designed with Primer express 2.0.0 (Applied Biosystems) on the region which covered all transcripts and overlapping exons if possible. Two multiplex sets were designed and are described in the Additional files (see Additional files 3 & 4: Table 3 & 4). The gene expression analysis was performed according to the manufacturer descriptions. The gene expression levels were measured using the Applied Biosystem 7500 Real-Time PCR system by reverse transcription (20 min, 50 °C), polymerase activation (15 min, 95 °C), and 2-step cycling (30 s at 95 °C, 75 s at 60 °C). The > 0.32 log_2_ fold change was used as a cut-off value because this fold change is the detection limit of qPCR using Taqman probes.

### Statistical analysis

Sequence data analysis was performed by setting the cycle threshold (Ct) per individual gene. The ΔCt was calculated by subtracting the Ct value of GAPDH from the Ct value of the gene. The -ΔΔCt was calculated by subtracting the Ct value control sample from unstimulated cells from the ΔCt value of the gene. Finally, for the relative gene expression levels, data were log-transformed (2^-ΔΔCt^), and were compared with the RNA-seq results. Fold change differences between control and target gene are presented as mean ± SD. Results were analysed using GraphPad Prism 8.3 (GraphPad Software, La Jolla, CA, USA). Data from legume protein incubations were evaluated by an independent t-test or by one-way ANOVA followed by Tukey’s multiple comparison test. A *p*-value of < 0.05 was considered statistically significant. The Venn diagrams were drawn using software from www.meta-chart.com.

## Supplementary Information

**Additional file 1: Table 1.** 64 DEGs shared by the two comparisons of weakly and strongly allergenic 2S albumins and 7S globulins. 64 genes that were differently expressed when PBMCs were incubated with a pair of weakly and strongly allergenic proteins from the 2S albumin protein family compared to a protein pair of weakly and strongly allergenic proteins from the 7S globulin protein family.

**Additional file 2: Table 2.** Source and protein family of proteins examined in the study. Overview table which includes the legumes from which the individual proteins were purified. In addition, the protein family is indicated and the classification if the protein is either a weakly or strongly allergenic protein is indicated.

**Additional file 3: Table 3.** Multiplex gene set 1. Table containing the sequences of the primers and probes of CCL2 and IL-24 designed with primer express software (Applied Biosystems).

**Additional file 4: Table 4.** Multiplex gene set 2. Table containing the sequences of the primers and probes of CCL7 and RASD2 designed with primer express software (Applied Biosystems).

**Additional file 5: Figure 1.** The immunological roles of CCL2, CCL7 and RASD2 in mast cell activation and food allergy. Figure explaining the immunological roles of CCL2, CCL7 and RASD2 in mast cell activation and food allergy.

## Data Availability

RNA-seq data in this publication have been deposited in NCBI’s Gene Expression Omnibus and are accessible through GEO series accession number GSE169031.

## Abbreviations

ANOVA: Analysis of variance
Ct: Cycle threshold
DEG: Differentially expressed genes
FCS: Foetal calf serum
GAGE: Generally applicable gene-set enrichment
IgE: Immunoglobulin E
IL: Interleukin
LPS: Lipopolysaccharide
PBMC: Peripheral blood mononuclear cell
RNA-seq: RNA-sequencing
qPCR: Real Time Quantitative PCR
SD: Standard deviation
