# Supplementary material for: Predicting the allergenicity of legume proteins using a PBMC gene expression assay
Source: BMC Immunol. 2021 Apr 13;22:27. doi: 10.1186/s12865-021-00415-x (PMC8042678; doi:10.1186/s12865-021-00415-x)
Supplement: Supplementary file 1 — Additional file 1: Table 1. 64 DEGs shared by the two comparisons of weakly and strongly allergenic 2S albumins and 7S globulins. 64 genes that were differently expressed when PBMCs were incubated with a pair of weakly and strongly allergenic proteins from the 2S albumin protein family compared to a protein pair of weakly and strongly allergenic proteins from the 7S globulin protein family. [file 12865_2021_415_MOESM1_ESM.docx]

**Additional Table 1:** 64 DEGs shared by the two comparisons of weakly and strongly allergenic 2S albumins and 7S globulins.

| **Gene ID** | **Gene name** | **Gene description** |
| --- | --- | --- |
| ENSG00000100302 | RASD2 | RASD family member 2 |
| ENSG00000153162 | BMP6 | Bone morphogenetic protein 6 |
| ENSG00000103888 | CEMIP | Cell migration inducing hyaluronidase 1 |
| ENSG00000136379 | ABHD17C | Abhydrolase domain containing 17C |
| ENSG00000112394 | SLC16A10 | Solute carrier family 16 member 10 |
| ENSG00000165474 | GJB2 | Gap junction protein beta 2 |
| ENSG00000204020 | LIPN | Lipase family member N |
| ENSG00000162892 | IL24 | Interleukin 24 |
| ENSG00000121743 | GJA3 | Gap junction protein alpha 3 |
| ENSG00000146072 | TNFRSF21 | TNF receptor superfamily member 21 |
| ENSG00000150510 | FAM124A | Family with sequence similarity 124 member A |
| ENSG00000135111 | TBX3 | T-box 3 |
| ENSG00000171049 | FPR2 | Formyl peptide receptor 2 |
| ENSG00000105509 | HAS1 | Hyaluronan synthase 1 |
| ENSG00000093134 | VNN3 | Vanin 3 |
| ENSG00000120875 | DUSP4 | Dual specificity phosphatase 4 |
| ENSG00000146374 | RSPO3 | R-spondin 3 |
| ENSG00000152784 | PRDM8 | PR/SET domain 8 |
| ENSG00000198682 | PAPSS2 | 3'-phosphoadenosine 5'-phosphosulfate synthase 2 |
| ENSG00000122641 | INHBA | Inhibin subunit beta A |
| ENSG00000171174 | RBKS | Ribokinase |
| ENSG00000112303 | VNN2 | Vanin 2 |
| ENSG00000099985 | OSM | Oncostatin M |
| ENSG00000013619 | MAMLD1 | Mastermind like domain containing 1 |
| ENSG00000198814 | GK | Glycerol kinase |
| ENSG00000221869 | CEBPD | CCAAT enhancer binding protein delta |
| ENSG00000142224 | IL19 | Interleukin 19 |
| ENSG00000172594 | SMPDL3A | Sphingomyelin phosphodiesterase acid like 3A |
| ENSG00000140519 | RHCG | Rh family C glycoprotein |
| ENSG00000106341 | PPP1R17 | Protein phosphatase 1 regulatory subunit 17 |
| ENSG00000123689 | G0S2 | G0/G1 switch 2 |
| ENSG00000196878 | LAMB3 | Laminin subunit beta 3 |
| ENSG00000170312 | CDK1 | Cyclin dependent kinase 1 |
| ENSG00000145632 | PLK2 | Polo like kinase 2 |
| ENSG00000052795 | FNIP2 | Folliculin interacting protein 2 |
| ENSG00000002587 | HS3ST1 | Heparan sulfate-glucosamine 3-sulfotransferase 1 |
| ENSG00000101188 | NTSR1 | Neurotensin receptor 1 |
| ENSG00000079308 | TNS1 | Tensin 1 |
| ENSG00000122254 | HS3ST2 | Heparan sulfate-glucosamine 3-sulfotransferase 2 |
| ENSG00000114268 | PFKFB4 | 6-phosphofructo-2-kinase/fructose-2,6-biphosphatase 4 |
| ENSG00000100628 | ASB2 | Ankyrin repeat and SOCS box containing 2 |
| ENSG00000064225 | ST3GAL6 | ST3 beta-galactoside alpha-2,3-sialyltransferase 6 |
| ENSG00000143344 | RGL1 | Ral guanine nucleotide dissociation stimulator like 1 |
| ENSG00000140465 | CYP1A1 | Cytochrome P450 family 1 subfamily A member 1 |
| ENSG00000164266 | SPINK1 | Serine peptidase inhibitor, Kazal type 1 |
| ENSG00000160791 | CCR5 | C-C motif chemokine receptor 5 (gene/pseudogene) |
| ENSG00000177575 | CD163 | CD163 molecule |
| ENSG00000232810 | TNF | Tumor necrosis factor |
| ENSG00000122877 | EGR2 | Early growth response 2 |
| ENSG00000178789 | CD300LB | CD300 molecule like family member b |
| ENSG00000179163 | FUCA1 | Alpha-L-fucosidase 1 |
| ENSG00000163823 | CCR1 | C-C motif chemokine receptor 1 |
| ENSG00000089041 | P2RX7 | Purinergic receptor P2X 7 |
| ENSG00000225107 | AC092484.1 | Novel transcript |
| ENSG00000234191 | AC091808.1 | Novel transcript |
| ENSG00000004799 | PDK4 | Pyruvate dehydrogenase kinase 4 |
| ENSG00000255491 | AC100858.3 | Novel transcript |
| ENSG00000106366 | SERPINE1 | Serpin family E member 1 |
| ENSG00000126262 | FFAR2 | Free fatty acid receptor 2 |
| ENSG00000100311 | PDGFB | Platelet derived growth factor subunit B |
| ENSG00000134780 | DAGLA | Diacylglycerol lipase alpha |
| ENSG00000108688 | CCL7 | C-C motif chemokine ligand 7 |
| ENSG00000135929 | CYP27A1 | Cytochrome P450 family 27 subfamily A member 1 |
| ENSG00000108691 | CCL2 | C-C motif chemokine ligand 2 |

# Gene descriptions were obtained from the National Center for Biotechnology Information (https://www.ncbi.nlm.nih.gov/gene).
