# Supplementary material for: Predicting the allergenicity of legume proteins using a PBMC gene expression assay
Source: BMC Immunol. 2021 Apr 13;22:27. doi: 10.1186/s12865-021-00415-x (PMC8042678; doi:10.1186/s12865-021-00415-x)
Supplement: Supplementary file 2 — Additional file 2: Table 2. Source and protein family of proteins examined in the study. Overview table which includes the legumes from which the individual proteins were purified. In addition, the protein family is indicated and the classification if the protein is either a weakly or strongly allergenic protein is indicated. [file 12865_2021_415_MOESM2_ESM.docx]

**Additional Table 2:** Source and protein family of proteins examined in the study.

| **Source** | **Protein family** | **Name** | **Weakly or strongly**  **allergenic protein?** |
| --- | --- | --- | --- |
| Lupine | 2S albumin | δ-conglutin | Weak |
|  | 7S globulin | Lup an 1 | Strong |
|  | 11S globulin | α-conglutin | Weak |
| Green pea | 11S globulin | Legumin A | Weak |
| Soybean | 7S globulin | Gly m 5 | Strong |
|  | 11S globulin | Gly m 6 | Strong |
| Peanut | 2S albumin | Ara h 2 | Strong |
|  | 7S globulin | Ara h 1 | Strong |
| White bean | 7S globulin | Phaseolin | Weak |
|  | 11S globulin | Legumin | Weak |
