# Supplementary material for: Predicting the allergenicity of legume proteins using a PBMC gene expression assay
Source: BMC Immunol. 2021 Apr 13;22:27. doi: 10.1186/s12865-021-00415-x (PMC8042678; doi:10.1186/s12865-021-00415-x)
Supplement: Supplementary file 3 — Additional file 3: Table 3. Multiplex gene set 1. Table containing the sequences of the primers and probes of CCL2 and IL-24 designed with primer express software (Applied Biosystems). [file 12865_2021_415_MOESM3_ESM.docx]

**Additional Table 3:** Multiplex gene set 1.

| **Multiplex set 1** | **Sequence** |
| --- | --- |
| CCL2-F | CAGCCAGATGCAATCAATGC |
| CCL2-probe | NED-CCAGTCACCTGCTGTTA-MGB |
| CCL2-R | GCACTGAGATCTTCCTATTGGTGAA |
| IL-24-F | GGCCATGGGTCCCATTCT |
| IL-24-probe | FAM-CAGGATTATTGTCAAAGAAGT-MGB |
| IL-24-R | TGTCACTGGCGCTGCTTAAA |
| GAPDH-F | GTCATGGGTGTGAACCATGAGA |
| GAPDH-probe | VIC-ACAGCCTCAAGATC-MGB |
| GAPDH-R | GGTGCAGGAGGCATTGCT |
