# Supplementary material for: Predicting the allergenicity of legume proteins using a PBMC gene expression assay
Source: BMC Immunol. 2021 Apr 13;22:27. doi: 10.1186/s12865-021-00415-x (PMC8042678; doi:10.1186/s12865-021-00415-x)
Supplement: Supplementary file 4 — Additional file 4: Table 4. Multiplex gene set 2. Table containing the sequences of the primers and probes of CCL7 and RASD2 designed with primer express software (Applied Biosystems). [file 12865_2021_415_MOESM4_ESM.docx]

**Additional Table 4**: Multiplex gene set 2

| **Multiplex set 2** | **Sequence** |
| --- | --- |
| CCL7-F | TGGATTTTGGTGGGTTTTGAA |
| CCL7-probe | NED- ATAAAGCCTTGGATGTATATG- MGB |
| CCL7-R | CCACAGTTTTTACAGCACTGAGATG |
| RASD2-F | CCACCATCGAGGACTTCCA |
| RASD2-probe | FAM- AGGTATACAACATCCGCG- MGB |
| RASD2-R | GGATGTCGAGCTGGTACATGTC |
| GAPDH-F | GTCATGGGTGTGAACCATGAGA (same as set 1) |
| GAPDH-probe | VIC-ACAGCCTCAAGATC-MGB (same as set 1) |
| GAPDH-R | GGTGCAGGAGGCATTGCT (same as set 1) |
