# Supplementary figures and images for: Predicting the allergenicity of legume proteins using a PBMC gene expression assay
Source: BMC Immunol. 2021 Apr 13;22:27. doi: 10.1186/s12865-021-00415-x (PMC8042678; doi:10.1186/s12865-021-00415-x)

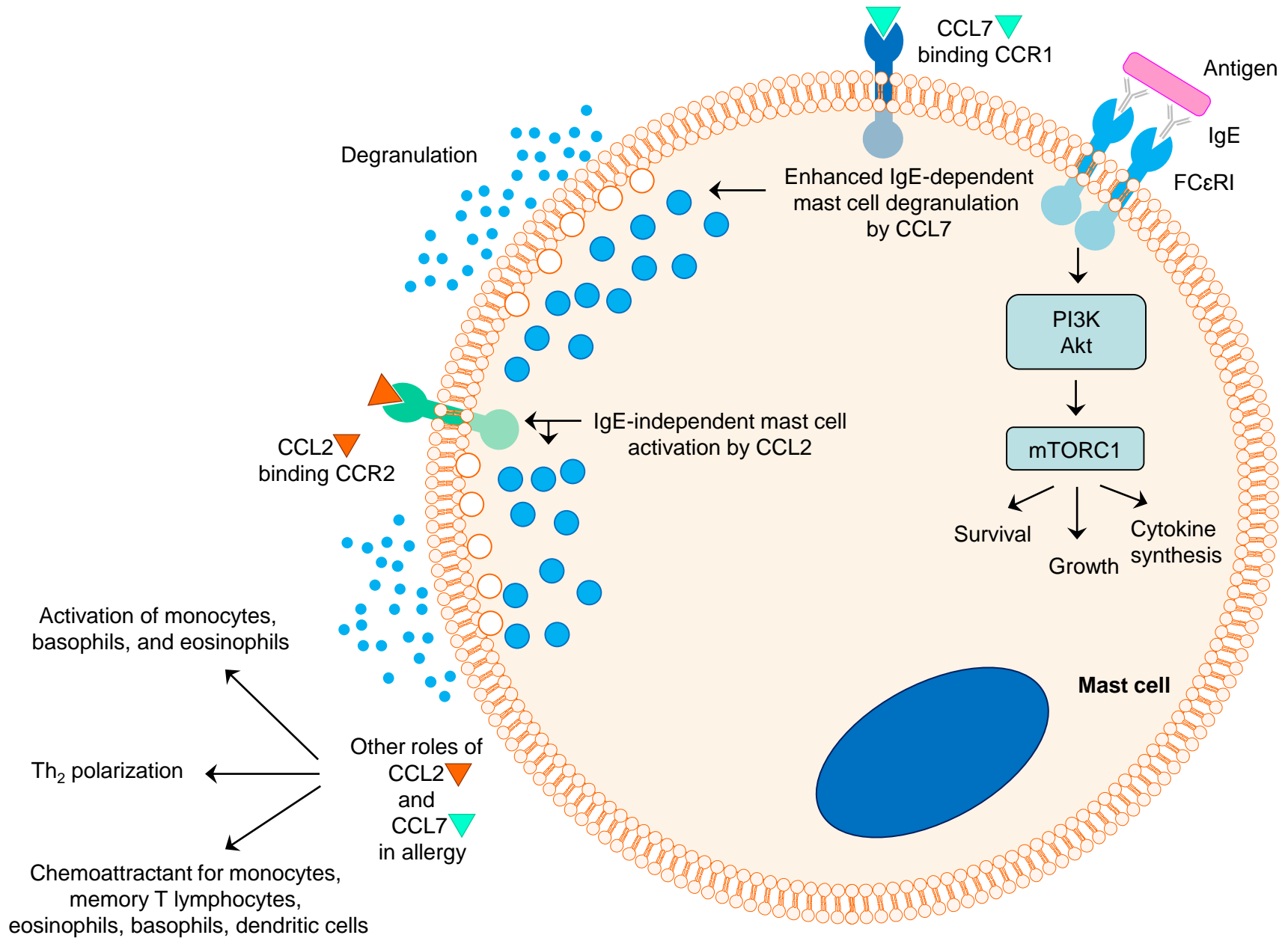

Supplement: Supplementary file 5 — Additional file 5: Figure 1. The immunological roles of CCL2, CCL7 and RASD2 in mast cell activation and food allergy. Figure explaining the immunological roles of CCL2, CCL7 and RASD2 in mast cell activation and food allergy. [file 12865_2021_415_MOESM5_ESM.pdf]
